# Addiction as a disorder of belief

**DOI:** 10.1007/s10539-014-9434-2

**Published:** 2014-02-23

**Authors:** Neil Levy

**Affiliations:** Florey Institute of Neuroscience and Mental Health, University of Melbourne, Parkville, 3010 Australia

**Keywords:** Addiction, Prediction errors, Mesolimbic dopamine system, Belief

## Abstract

Addiction is almost universally held to be characterized by a loss of control over drug-seeking and consuming behavior. But the actions of addicts, even of those who seem to want to abstain from drugs, seem to be guided by reasons. In this paper, I argue that we can explain this fact, consistent with continuing to maintain that addiction involves a loss of control, by understanding addiction as involving an oscillation between conflicting judgments. I argue that the dysfunction of the mesolimbic dopamine system that typifies addictions causes the generation of a mismatch between the top-down model of the world that reflects the judgment that the addict ought to refrain from drugs, and bottom-up input caused by cues predictive of drug availability. This constitutes a powerful pressure toward revising the judgment and thereby attenuating the prediction error. But the new model is not stable, and shifts under the pressure of bottom-up inputs in different contexts; hence the oscillation of all-things-considered judgment. Evidence from social psychology is adduced, to suggest that a similar process may be involved in ordinary cases of weakness of will.

Addicts, it is widely held, suffer from some kind of problem with their ability to act as they judge they ought. Though some thinkers have denied this claim—holding that addicts simply have different preferences to the rest of us—the view that addicts suffer from some kind of deficit with regard to action control seems to have the vast bulk of evidence in its favor. But how should we understand the loss of control involved in addiction? In this paper, I shall argue that addicts lose control over their actions by losing control, first, of their beliefs: they shift from judging they ought to abstain from drugs to judging that they ought (all things considered) consume on this occasion. But this shift is not a mere change of mind; it is not a rational response to evidence and it does not express the addicts’ settled preferences. Rather, it is the result of a dysfunctional reward valuation system. The evidence I shall cite for this claim comes in several forms, but the bulk of the evidence comes from neuroscience. I shall argue that the (admittedly controversial and still somewhat speculative) hypothesis that the brain is a prediction error minimization machine supports the case for a dysfunctional oscillation in addicts’ judgments.

## Loss of control in addicts

Following the lead of jurists, we might distinguish cognitive and volitional sources of disordered action. Many legal systems recognize two kinds of partial or total defenses turning on mental illness or dysfunction. The less controversial kind of excuse is cognitive, and is enshrined in the famous M’Naughten rules. Roughly, on the cognitive prong of a mental incapacity test, an agent is found not guilty if (a) he or she did not understand the nature of the action they performed, or, if they did understand its nature, did not understand that it was wrong, *and* (b) these failures of understanding were due to a mental illness. The cognitive prong is more or less universally recognized as a defense in law (though juries often seem reluctant to utilize it in their decision-making). Many jurisdictions recognize only a cognitive defense, but some jurisdictions also recognize a volitional defense. Accused persons can avail themselves of a volitional defense if they do not suffer from a (relevant) defect in reasoning but are unable to act as they judge they ought.

Both laypeople and scientists commonly understand addiction as involving some kind of volitional failure. Drug taking is widely held to become compulsive in addiction (see, among many other instances, Leshner 1999; Hyman 2005; Everitt and Robbins 2005; Charland 2012), where ‘compulsion’ is cashed out in terms of an irresistible desire overcoming the agent despite her better judgment. In this paper, I will argue in support of a rival minority view, according to which the failure of agency it involves is better thought of as cognitive than volitional (though distinct from the kind of cognitive failure recognized by the M’Naughten laws). According to this rival view, the best-known proponent of which is George Ainslie (2001), the pathology of self-control from which addicts suffer does not involve agents acting contrary to their own better judgments. At the time of action, and (on my version of this view, though not Ainslie’s) typically for long stretches of time prior to it, addicts actually judge that consuming the drug is, all things considered, the best option available to them. Addicts are not compelled by desires, cravings or other motivational states to act contrary to their own best judgments at the moment they act; rather, addicts (who may sincerely and wholeheartedly judge most of the time that they ought not to consume drugs), find themselves judging that they ought, all things considered, to consume, and act accordingly (where ‘acting accordingly’ may involve a long series of actions aimed at procuring the drug and the opportunity to take it).

One way to argue for this kind of claim is by reference to behavior. Consider the series of actions involved in getting an agent to the point at which he or she takes an illegal drug. She may have to find the money for drugs (which could involve going to the bank or could involve burglary), seek out a dealer, drive to the location, buy the drugs, drive to a secluded place where she can prepare the drugs and only then, finally, take them. It might be possible that an agent could be compelled—say by an irresistible desire—to take a drug, but it is intuitively implausible that she should be compelled to engage in this long series of actions.

Further, the series of actions in which addicts engage in order to reach the stage at which they can consume their drug is not merely too extended and too complex to plausibly be regarded as compelled by an irresistible desire; it also seems too sensitive to the kinds of incentives and disincentives that normally modulate action to be compelled. Addicts do not just refrain from consuming drugs when a policeman is at their elbow. They also plan the entire series of actions more or less carefully, in some cases carrying out schemes of great ingenuity to procure money for drugs. Moreover, the majority of addicts eventually give up taking drugs, and they seem to do so in response to perfectly ordinary incentives (Heyman 2009). It is when sufficiently rewarding alternatives with which drug-taking competes are available to them that they finally succeed in giving up for good.

All of this evidence seems to indicate that the actions of addicts are responsive to *reasons*, in the way actions that are controlled by agents’ judgments ought to be. In turn, this seems to indicate that addicts act as they do because they judge that so doing is, of all the options available to them, best. It is this evidence that is cited by those few scholars who think that addicts simply have unusual preferences: they want to take drugs, and do not suffer from any sort of incapacity at all (Foddy and Savulescu 2010). But the same sort of evidence which is cited in support of the view that addicts’ behavior is regulated by some kind of judgment-like state the content of which involves an endorsement of their drug-taking can be cited in support of the view that the very same addicts *also* have a judgment-like state with a conflicting content. We can point, for instance, to the time and energy they invest in series of actions designed to avoid drug taking (Ross et al. 2008). The behavioral evidence seems to support attribution of conflicting judgments to addicts. As we shall see, I believe that in fact the appearances here are not misleading: it is appropriate to attribute both states to addicts, though not simultaneously. The behavioral evidence is best interpreted as indicating that addicts suffer from some kind of incapacity or deficit that causes them to oscillate from one all things considered judgment to another, inconsistent with the first.

A view like this is not unprecedented: though very much a minority view, the claim that the proximate cause of loss of control is judgment-shift has been forcefully urged by George Ainslie (2001). Ainslie claims that agents lose control because we are hyperbolic discounters; as a consequence, the value of immediate rewards climbs steeply for us, and our discount curves for future goods can cross. At least as an explanation of loss of self-control in addiction, Ainslie’s view is highly controversial, with most contemporary accounts remaining committed to the claim that addiction centrally (if not exclusively) involves some kind of volitional failure (Schroeder and Arpaly 2013; Holton and Berridge 2013). Ross (2013) argues that Ainslie ignores the neuropsychological differences between ordinary agents and addicts; whatever the merits of his view as an explanation of ordinary weakness of will, it cannot account for the more spectacular pathologies of action seen in addiction, he claims. Contra Ross, I will argue that the neural adaptations characteristic of addiction provide a mechanism for judgment shift of the general kind Ainslie describes (though I do not take myself to be committed to all the features of Ainslie’s view: in particular, on my account cues predictive of drug availability cause preference-reversals as effectively as immediately available rewards). Providing a credible neural mechanism not only helps to defuse the criticism that accounts like Ainslie’s ignore the neuropsychological differences between addicts and ordinary agents, it also increases the overall plausibility of judgment-shift based accounts of loss of control more generally.

In what follows, I will argue that neural adaptations central to substance addiction cause addicts to undergo an oscillation in their judgments. More specifically, I shall claim that addicts are prone to experience cue-dependent judgments: addicts who judge that they ought to *A* at *t*, because *A*-ing best achieves their goals, shift to judging that they ought to perform some incompatible action at *t*1 in response to cues predictive of drug availability. Since the change of judgment is not one that occurs in response to evidence (or at any rate, not in response to evidence that the agent was not aware of or failed to take account of at *t*, in forming the judgment that they ought to *A*), the change of mind is best thought of as involving a loss of control, rather than as the product of mechanisms for the rational guidance of behavior.

I will also adduce some evidence from social psychology, this time concerning ordinary agents rather than addicts. It is possible that the mechanisms involved in addiction are quite different to those involved in ordinary failures of control. However, I shall suggest that in the light of theoretical considerations, it is likely that a similar kind of process occurs both in addicts who experience cravings that lead them to consumption and in ordinary agents who find themselves confronted with, and ultimately giving into, temptations. Let me begin by sketching these theoretical considerations.

## The hypothesis testing brain

Many scientists now believe that the brain is basically a Bayesian hypothesis tester. One of its primary activities is making predictions about what input it expects to receive and then updating these predictions in the light of the actual input. The brain attempts to minimize prediction error (the component of the input that is not predicted by the brain’s current model). This kind of process occurs at many different levels of the processing hierarchy within the brain, with the outputs of lower levels feeding into, and being constrained by, higher levels, and encompassing many different processes, from perception to belief formation to learning.

Though any divergence between expected inputs and actual inputs is utilized by the brain to update its predictions, prior predictions constrain the interpretation of bottom-up input. The prior models the brain uses to generate its predictions shape the perception of the inputs used to adjust these same models. Constraining what is perceived in light of the expected input is obviously adaptive under a range of circumstances. First, it allows the brain to make sense of degraded or noisy inputs. We may smoothly fill in gaps in speech or noise in incoming visual information in ways that allow us to respond to the inputs efficiently. Second, it allows for rapid response: the organism can react to its predicted model of how the world unfolds prior to receiving inputs that confirms the prediction. Third, it is often better to act on models of the world held with high confidence, even if those models are (somewhat) wrong then to be paralyzed by uncertainty. But if error is not trivial, we need a mechanism for minimizing it. Brains work to minimize this error. They do this at every level of organization, from neural firing rates to synaptic connectivity to the motivation of behavior aimed at information gathering (e.g. visual saccades).

Many different psychological processes can be explained by reference to the uses and transformations of prediction errors. Learning might best be understood as the revision of a top-down model in the light of a prediction error. Perception might be seen as the currently best prediction of expected input. And so on. It is important to see that this process is winner takes all: only one model wins out and others are inhibited (Hohwy 2010).

I shall argue that the loss of control seen in addiction might best be understood as caused by a judgment shift as the brain seeks the best fit between top-down models and bottom-up sensory input. In order to prepare the ground for this hypothesis, it might be helpful to see how the prediction error model of belief formation can be used to explain pathologies of belief, such as delusions. A delusional belief is a false belief, often with a bizarre content, held in the face of overwhelming evidence and which is not explained by the person’s social or cultural background (so the beliefs of, say, Raëlians may not count as delusions). Delusions may arise subsequent to brain lesion; this is the common etiology of delusions like Capgras’ delusion, which is characterized by the belief that familiar people (typically spouses, children and other relatives) and occasionally even animals have been replaced with impostors, and the much rarer Cotard’s delusion (the delusion that the person herself is dead). Other delusions, like delusions of alien control and thought insertion, are first-rank symptoms of schizophrenia.^1^

Many cognitive scientists accept a two factor account of delusions (Davies et al. 2001; Stone and Young 1997). Factor one is typically held to be an abnormal experience, consequent on brain insult or disease. For instance, factor one in the formation of Capgras delusion might be a brain abnormality that strips away the normal feeling of familiarity accompanying perception of a loved one. The deficit is presumed to be the result of a dysfunction in the dorsal perceptual stream, which is (largely) unconscious (Capgras is therefore the mirror image of prosopagnosia, in which patients have difficulty in recognizing faces; in prosopagnosia, the ventral processing stream is hypoactive, but the dorsal stream may be normal; hence prosopognosics may exhibit normal signs of autonomic arousal in response to familiar faces; Ellis and Lewis 2001). However, two factor theorists argue that the first, experiential, factor is insufficient to account for the delusion. They have two main reasons for thinking that there must be a second factor. First, it seems implausible that an anomalous experience could be sufficient to explain a bizarre belief, at least in a person who is globally rational (and delusional patients may seem quite rational in other respects). After all, the person may be entirely capable of understanding the fact that they have had a stroke (say), and apparently of understanding how a stroke might fully account for their unusual experience. Since they know that their belief is bizarre, and they know that the experience which inclines them toward it arises from processes that are not truth-tracking, it seems that mere anomalous experience is not sufficient to explain why they nevertheless go on to form the delusional belief. Second, some patients who apparently have the kind of anomalous experiences hypothesized to be partial causes of delusions do not go on to form delusional beliefs. It is the second factor, two factor theorists maintain, that distinguishes these patients from those who suffer from delusions.

The second factor is typically held to be some kind of deficit in reasoning. There is indeed some evidence for reasoning deficits in delusional patients. For instance, schizophrenics seem to have a tendency to form beliefs on the basis of evidence that normal controls judge to be insufficient. In one experiment, deluded patients and normal controls were shown balls drawn from an urn one by one. They were informed that the balls were drawn from one of two urns—one of which contains 80 % blue balls and one of which contains 80 % red balls—and asked which urn the balls come from. Delusional patients were significantly quicker to form a judgment as to which urn the balls come from (i.e., required significantly fewer balls to form a judgment) and expressed relatively high confidence in their judgment, compared to normal controls (Bentall et al. 1991). Further, once they have formed a belief, suffers from delusions apparently require significantly more disconfirming evidence to change their minds (Woodward et al. 2008).

However, the evidence that delusional patients have deficits in reasoning is controversial, and many of the predictions to which two factors accounts seem to be committed are not well supported. These accounts predict, for instance, that patients should form false beliefs in response to visual illusions, but this seems not to be the case (Hohwy and Rajan 2012). Problems like these have led some researchers to think that we need a different style of explanation of delusions. Hohwy (2010; Hohwy and Rajan 2012) has suggested that the prediction error model of belief formation can elegantly explain pathologies of belief formation, such as delusional belief, without needing to postulate a deficit in reasoning.

Hohwy argues that delusions can be explained by a single, experiential, factor. On his model, the anomalous experience postulated by two factor theories is reconceived as a very large prediction error; delusional perception arises from the attempt to account for the error. Consider the delusion of alien control. The experiential factor here apparently arises from some kind of failure of the mechanisms involved in self-monitoring. In normal agency, an efference copy of motor activity is sent to comparator systems (Frith et al. 2000). A match between the sensory input predicted on the basis of this efference copy and actual sensory input leads to the attenuation of self-generated input and to a sense of agency. In schizophrenia, this mechanism is dysfunctional, as evidenced by a decrease in attenuation of self-generated input (schizophrenics, unlike normal controls, can tickle themselves; Blakemore et al. 2000). Due to the dysfunction in the comparator system, schizophrenics lose a sense of agency over their actions and even over their thoughts. On a two factor account of delusions, this absence of normal experience provides the first factor in explaining auditory hallucinations (which are partially explained by an absence of the experience of agency over subvocalizations); thought insertion and alien control. But Hohwy suggests that mismatchs like these may be sufficient to explain delusions by themselves.

For Hohwy, the delusion of alien control arises from the brain’s attempts to minimize the large prediction error that the anomalous input constitutes. A model must be found to account for the prediction error. Only some kind of supernatural model has the right kind of features to accommodate the temporal dynamics of the experience; rival models (such as the neuroscientifically-informed brain dysfunction model) do not generate predictions at the rapid time scales of processing of sensorimotor systems. As a consequence, the content of the supernatural agency models comes to be embedded in the perceptual experience. The delusional patient *experiences* her movement as controlled by outside forces; she does not *interpret* her movement as so controlled.^2^ The content that is perceptually embedded is, if not quite a full blown belief, at least a doxastic state of the agent (indeed, Hohwy suggests that the distinction between perception and belief is merely one of time scale; Hohwy and Rajan 2012).

## Addiction and judgment oscillation

It must be conceded that both the Bayesian brain theory and—a fortiori—the explanation of delusional beliefs that builds on it remain somewhat speculative. The Bayesian brain hypothesis was initially a computational hypothesis: it was speculated that the brain might be a prediction error detection machine because the hypothesis provided an elegant algorithm for learning. The hypothesis acquired empirical grounding when it was discovered that mechanisms in the brain appear to actually encode the requisite algorithms in their firing patterns. Many scientists are now convinced that the Bayesian brain hypothesis provides the key to a unified theory of cognitive processes (see Clark 2013, for review). However, direct evidence that Bayesian updating is as pervasive as the theory requires remains patchy. In what follows, however, I shall assume both that the core theory and the account of delusions that Hohwy builds on it are (more or less) correct. On that basis, I will build my account of judgment oscillation in addicts.

Just as the prediction error produced by the faulty comparator system produces delusions of alien control, thought insertion and auditory hallucinations, and the prediction error produced by a dysfunction in the dorsal perceptual stream produces the Capgras delusion, I shall suggest, a prediction error—a mismatch between the top down model of the world and sensory input—causes judgment shift in addiction. Under the influence of cues that predict drug availability, which generate a large prediction error, addicts perceive pursuing drugs as the (all things considered) best option available to them. That judgment is perceptually embedded, in an analogous way to the way in which the judgment that < my thoughts are inserted by an outside agency > might be perceptually embedded for a schizophrenic: that is, the judgment is part of the content of the phenomenology of perception. Perceptual embedding of a judgment typically causes the agent to go on to form a personal-level belief with a matching content (the mechanisms that explain whether she does or does not go on to form this personal level belief will involve prediction error minimization at higher levels of processing). There are, I shall suggest, factors that powerfully bias the addict towards such a personal level belief. The belief state so generated is, however, unstable over time. Addicts experience a cue-driven oscillation between judgments, where the oscillation is itself explained by prediction errors.^3^

Addiction involves a range of brain alterations, including the dysregulation of reward circuits, increases in corticotropin-releasing factor and hypoactivity in the orbitofrontal–infralimbic cortex (see Koob and Le Moal 2008, for review). However, many researchers believe that pathological changes in the midbrain dopaminergic system are central to becoming and remaining addicted (Kalivas and Volkow 2005; Hyman 2005; Ross et al. 2008; Schroeder 2010; Yaffe 2013). Midbrain dopamine neurons send dense projections to the basal ganglia and prefrontal cortex. The precise role of the mesolimbic dopamine system remains disputed. However, it seems apparent that whatever its precise role, the system encodes some kind of prediction error.

In several experiments, Schultz et al. (1992) recorded the activity of midbrain dopamine neurons in monkeys performing various tasks that were rewarded with water or juice. In one experiment, monkeys learned that they would receive a reward if they pressed a particular lever following a cue (Schultz et al. 1992). During the learning phase, the neurons responded strongly to the delivery of the reward, but once the task, and the association between the cue and juice availability was learned, neurons responded when the cue was given, but not when the reward was delivered. Similarly, in later work dopamine neurons in monkeys responded initially to the delivery of a reward predicted by a visual cue, but as the association between the cue and the reward came to be learned, the response to the reward declined while the response to the cue predicting the reward increased (Sutton and Barto 1998).

Addiction centrally involves dysregulation in this same midbrain dopamine system. Almost all addictive drugs increase dopaminergic activity. Amphetamine, nicotine, cannabis, cocaine and alcohol all either stimulate dopamine release or decrease dopamine reuptake. They thereby increase dopamine in the nucleus accumbens. Opiods increase dopamine indirectly, by influencing neurons that alter accumbal dopamine (Carter and Hall 2012). The manner in which addictive drugs (and, in a very different way, gambling; see Ross et al. 2008) drive up the dopamine signal is widely thought to be central to explaining how addiction develops and why it is a chronic relapsing condition. Understanding the precise role of the mesolimbic dopamine system therefore seems essential for understanding addiction. For many addiction experts, addiction *is* a pathology of the dopaminergic system. In the common metaphor, addictive drugs ‘hijack’ this system.

Many researchers believe that the mesolimbic system is a reward prediction system (Montague et al. 1996; Schultz et al. 1997). On this view, dopamine neurons respond when an unexpected reward is delivered, or when a cue that strongly predicts a reward, but is itself unexpected, is delivered. The hypothesis that dopamine neurons in the midbrain play this role was strengthened by the finding that once monkeys learn the association between cue and reward, not only is doplaminergic response to the cue and not to the delivery of the reward, but there is actually a decrease below baseline in dopamine firing rates if the expected reward is withheld (Schultz et al. 1997). An increase in dopamine neuron firing rates indicates that an unexpected reward is available or will soon be available—that the world is better than expected—and a decrease in dopamine firing rates indicates that the world is worse than expected. This shows that dopamine firing rates are tracking reward prediction error, rather than reward itself.

The reward prediction hypothesis seems to explain addiction by understanding it as a pathology of reinforcement learning. When the system operates as it should, dopaminergic activation attenuates in response to *expected* reward. Dopamine response increases when the world is better than expected; when an expected reward is delivered, the world is exactly as expected and there ought to be no dopamine response. Given that drugs are extremely rewarding, we ought to expect an initial dopamine response to consumption, followed by attenuation of the dopamine response in further episodes of consumption as a result of habituation and a concomitant increase in dopamine response to predictors of drug availability. Instead, we find dopamine response to predictors of drug availability *and*—because drugs of addiction drive up the dopamine response by their chemical action—continuing dopaminergic activity at consumption as well. In effect, the dopaminergic system responds to drugs with the signal that consumption is better than expected, *every time* the drug is consumed. The addict cannot learn the reward value of a drug that directly drives up dopamine, because the system for reward value learning uses endogenous dopamine to track reward value. The result is pathological learning; the system treats the drug as of ever increasing value.

There are, it must be noted, plausible rivals to the reward prediction interpretation of mesolimbic dopaminergic activity. Berridge (2007) suggests that the role of dopamine is incentive salience, not learning. Berridge points out that learning about the relationship between a stimulus and a reward can occur without dopamine. In mice genetically engineered to be unable to synthesize dopamine, normal learning seems to occur. It also occurs in mice that have virtually no mesolimbic dopamine due to neurochemical lesioning. Further, activation in the ventral palladium, downstream of the mesolimbic dopamine system, is stronger in response to a second, redundant, predictor of reward than in response to the first. Since the second predictor adds no new information, we ought to expect a smaller response to the second predictor than to the first if the dopamine system was itself a reward prediction system.

For Berridge (2007; Holton and Berridge 2013), then, addiction is a pathology of incentive salience and not reward prediction. It does not involve pathological learning; rather it involves pathological ‘wanting’. I won’t try to adjudicate between these rival views. I shall argue that if we broaden the focus from the role of dopamine in addiction to its role in the brain more widely, we have good reason to think that the dopamine signal causes the addict to enter into a doxastic state: the addict sees the drug as good. I will first defend the hypothesis that dopamine subserves an error prediction system (and not merely a reward prediction mechanism); I shall then go on to suggest that the broad outlines of the view I defend are compatible with both accounts of the role of the mesolimbic dopamine system.

The mesolimbic dopamine system should be thought of as an error prediction mechanism, not merely a reward prediction mechanism because dopamingeric response results from the violation of expectations, regardless of whether there is any reward attached to the expectation. Corlett et al. (2004) measured a reliable signal of dopaminergic activity (rPFC activity^4^) in healthy subjects, as they learned about the association between events and their effects (they were asked to imagine that they were an allergist, advising a new patient who suffered reactions in response to some foods but not others; their task was to learn which foods predicted allergic reactions). A prediction error signal was observed in response to violations of expectations on the task. Given the role of prediction error seems to be to signal a discrepancy between actual sensory input and sensory input expected on the basis of the model of the world currently held to have the highest likelihood, we can expect that the role of the error signal will be to cause model update. And that is what we find: participants revise their associations, such that they no longer generate an error signal in response to results that formerly generated a prediction error.

Updating associations is updating our (very local) model of the world. It is updating the conception of what causes what or which properties are coinstantiated. It is important to note that prediction errors do not always cause an updating in beliefs, if by ‘beliefs’ we mean the mental states of folk psychology. Prediction errors may instead motivate the organism to act on the world to minimize the error. Action doesn’t require a change in beliefs, but it does require—at minimum—the updating of some kind of doxastic state. Acting to minimize a prediction error entails representing a gap between expected input and actual input; that representational state *is* a doxastic state, though it may be subpersonal and its contents may be too fragmented and encapsulated to count as a belief. Often, though, updating associations does cause an update of belief, whether personal-level beliefs that motivate action to minimize the prediction error or a longer-lasting belief which represents the world as having different properties than had been expected. Whether an organism adopts a belief of the latter sort or acts on the world will depend on the relative accessibility and costs of the options. In many circumstances, belief update is the only practical option (for instance, when the goal is learning about the world, as in the experiment described above). This generates a prediction of its own: when action to minimize prediction error is not accessible or too costly, anomalies in prediction error mechanisms will cause anomalous beliefs. This prediction seems to be borne out.

There is a great deal of evidence linking dysfunctions in the midbrain dopaminergic system to pathologies in belief formation. Corlett et al. (2007) repeated their study of rPFC activity in response to violations of learned associations, this time with healthy controls and patients with delusional beliefs. In the patient group, rPFC activity in response to violations of expectations was attenuated, while predicted events produced an abnormally high rPFC response. Further, the degree of abnormality in rPFC activity was significantly related to the severity of delusions in the patient group. The same research group found that magnitude of prediction-error response under placebo predicted likelihood of delusions under ketamine (Corlett et al. 2006). It seems likely that abnormalities in the midbrain dopaminergic system result in dysfunctions in the error prediction system, giving rise to aberrant beliefs. In other words, the mesolimbic dopamine system may be part of the mechanism that underlies Hohwy’s (2010) etiology of delusions via aberrant experiences.

Here is a sketch of delusion etiology: abnormalities in error prediction systems—including, but not limited to, the efference copy comparator system—lead to mismatches between sensory input and predicted input. The mismatch is surprising and therefore attention-grabbing. That is, a mismatch is attention-grabbing because it is unexpected. Indeed, attention might be thought of as a mechanism for optimizing precision in bottom-up signal (Hohwy 2012; Clark 2013): when we experience an unexpected mismatch, we pay attention so as to be able to detect more precisely what the mismatch consists in, and therefore how best we ought to respond to it (upregulation of attention may play a significant role in transforming subpersonal doxastic states into full-blown beliefs). It is widely held that the dopaminergic system is involved in attention regulation (Corlett et al. 2007; Fletcher and Frith 2009). In the monothematic delusions, the mismatch between predicted and actual input may be caused by a circumscribed lesion; in schizophrenia there may be a more widespread failure of the system (Corlett et al. 2007 mention how in the early stages of psychosis, patients report that many apparently trivial or irrelevant stimuli are attention-grabbing, which comports well with their finding that patients generate a larger than normal rPFC response to expected events). The patient is now motivated to reduce or eliminate the prediction error. There is no action he or she can take that would eliminate it (a Capgras sufferer can’t replace the apparent duplicate with his real wife). Instead, higher levels in the processing hierarchy search for an available model that will attenuate the prediction error. At an intermediate level of processing, the ‘as if’ content (it is *as if* someone is controlling my thoughts; it is *as if* my wife has been replaced by an impostor) is replaced by ‘face value’ content, because that content best explains the sensory input. Hence the patient comes to endorse the perceptual seeming that his thoughts are being controlled or that his wife has been replaced. At a higher level still, this content is elaborated in terms of culturally available models: God, or the CIA, is controlling my thoughts; my wife has been replaced by a robot. The causal route is from ‘as if’ content that is perceptually embedded, to endorsement by higher-level mechanisms on the basis of best fit with available models, to elaboration into world view.

I suggest that the abnormalities in the midbrain dopaminergic system caused by neuroadaptations to drug use cause analogous processes. Addiction involves *cue*-*dependent belief oscillations*. Here is a sketch of the causal mechanism:

Just as cues predictive of natural rewards come to be reliable elicitors of dopamine response, so cues predictive of drug availability are reliable elicitors of dopamine response in addicts. This helps to explain why relapse is often triggered by cues of drug availability (Carter and Hall 2012). As we would expect, given that dopamine response upregulates attention, drug-related cues are attention grabbing for addicts (Robinson and Berridge 2003). Given that greater attention is hypothesized to increase the gain on the prediction-error, however, the error is passed on to higher-levels of the processing hierarchy, which search for way to eliminate the prediction error: either by acting on the world or by updating the reigning causal model. In most cases, there will be no easily accessible action that minimizes the error, but there will be an available model of the world: the model of the world, and of the drug’s place in the world, which the person endorsed in the earlier stages of drug use when drug use was controlled and chosen for its rewards. Given that there is an available model that attenuates the prediction-error, there is powerful pressure for the model to be adopted and endorsed. Accordingly, the addict shifts from judging that they ought (all things considered) abstain from drug use to judging that drugs are worth taking for their hedonic reward (or some analogous belief content).

However, the belief is not stable. It persists so long as it best matches sensory input, but once the drug is consumed, or once the person is removed (for a sufficiently long period) from the influence of cues predicting drug availability, competing sensory input generate a new prediction-error. These sensory inputs may be generated by awareness of the satisfactions available from competing activities. Alternatively, a rival model of the world—one in which the addict is committed to staying drug free and avoiding the costs associated with consumption—may come to be triggered; this model may modulate bottom-up input such that a better match between model and input is achieved (given that different processes compete for access to central systems, anything which alters the salience of various options may powerfully influence which causal model of the world comes to be adopted by the organism; cues predicting the availability of opportunities to consume are highly salient for addicts but sufficient distance from such cues will result in rapid dwindling of their power to influence cognition). The addict oscillates back to believing that it is better to remain drug free.

On the perspective sketched here, the addict’s belief landscape is sculpted by two powerful attractors. These competing models each best explain sensory input under a wide range of conditions. Each is stable enough to persist for hours at a time, and to drive action that the model rationalizes. But unless the addict is removed from an environment in which cues associated with one of the rival models are encountered, neither is stable enough to remain in place. The result is oscillation between conflicting beliefs.

So far I have proceeded under the hypothesis that mesolimbic dopaminergic activity is part of an error prediction system. As we saw, some researchers reject this view. They point to the fact the dopamine system does not seem to be required for learning, and to the fact that it seems to play a direct role in motivating behavior in ways that seem independent of what the organism believes (Holton and Berridge 2013). I shall now suggest that these (plausible) claims are actually compatible with the broad outlines of the view sketched here.

The key to seeing how a dysfunctional dopaminergic system may play a role in pathological belief formation even if it does not constitute an error prediction system is to note that the dysfunction in error prediction in delusions lies outside the dopamine system itself. As we saw, in Capgras the mismatch between bottom up sensory input and top down expectations occurs because of a dysfunction of the dorsal perceptual system, rather than the dopamine system. Similarly, in delusions of control, thought insertion and auditory hallucinations, the mismatch results from a dysfunction of the action monitoring system, not the dopamine system. Nevertheless, there is good evidence that activity in the dopamine system is dysregulated in these pathologies.

In addiction, too, it may be that the prediction error does not directly arise from the mesolimbic dopamine system itself. Indeed, that is precisely Berridge’s view: he holds that the dopamine system *encodes* but does not *cause* the prediction error. It encodes a prediction error that arises somewhere else in the brain, whether in the dorsal perceptual system, the action monitoring system, or elsewhere.

What, then, is the role of the dopamine system in belief formation? Its most direct role may be, as Berridge thinks is generally true, motivational. It may motivate the organism to correct the prediction error it encodes. As we have already seen, there are two ways in which the prediction error may be corrected: world to mind or mind to world. That is, a mismatch between expectation and sensory input may be corrected by changing doxastic states (a mind to world direction of change) or by changing the sensory input by way of manipulating the world. Dopamine, which encodes a prediction error, may have its most direct role in motivating the organism to change *either* the world or its expectations about the world. Rather than *being* the prediction error mechanism itself, it may be downstream of the prediction error mechanism. Indeed, this hypothesis comports well not only with Berridge’s claims that the mesolimbic dopamine system is an incentive salience system, but also with the fact that we have already identified distinct error prediction systems in delusions. Relative accessibility of top down models and actions that will alter sensory input probably explain which wins out in the competition for access to central systems.^5^

The account of addiction offered here not only provides a unified explanation of the behavior seen in addicts, it also provides an explanatory framework that makes sense of available data concerning eventual recovery. In particular, it explains the striking fact that addiction seems relatively easy to beat when addicts are removed from environments in which they are likely to encounter cues associated with consumption. Few of the thousands of GIs returning from Vietnam addicted to heroin continued to use in the United States; most appear to have had relatively little difficulty in giving up (Loewenstein 2000). The account developed here explains this striking fact: the former GIs did not experience cue-driven judgment shift, because they encountered relevant cues rarely. The account predicts that treatment methods that remove addicts from cues associated with drug-taking will be particularly effective, at least in the short-term (many such treatments fail because the person is returned to their old neighborhood, and the old cues, upon leaving treatment). It also predicts that provision of competing distractions, if they are sufficiently attention-grabbing, will prevent cue-driven judgment shift. Flanagan (2013) argues that Alcoholics Anonymous works (not spectacularly, but at least as well as any other treatment) by offering very concrete distractions (for instance, mentors who one can call upon) and alternative social activities that compete with drinking.

## Evidence from social psychology

I have argued that the fact the mesolimbic dopamine system, which encodes prediction errors, is dysfunctional in pathologies of belief as well as addiction, together with the evidence that much of addicts’ behavior seems controlled by a doxastic state gives us some reason to think that addiction involves judgment oscillation. In this section, I will build on this—admittedly speculative—sketch of a mechanism for losing control of behavior via alterations in judgments. I will argue that the model receives very indirect support from social psychological work on self-control and its loss in normal subjects. If this claim is plausible, then the mechanism probably generalizes: it probably explains (some cases of) loss of control in ordinary subjects under ordinary conditions.

The social psychological evidence I have in mind comes from work on the so-called ego depletion hypothesis. The ego depletion hypothesis is the hypothesis that self-control depends on a depletable resource. The model is often compared to a muscle model of physical strength: just as the strength available for particular physical task is a function of use (long term use strengthens the muscle but recent use makes it weaker) so the resources available for self-control depend on past use. Recent engagement in demanding self-control tasks reduces self-control in the short term (though glucose seems to replenish resources), such that subjects who engage in successive self-control tasks do worse at the second task than controls who first perform a task that does not require self-control (Baumeister 2002; Baumeister and Vohs 2007).

Available evidence seems to suggest that loss of self-control in the ego depletion paradigm is mediated by an alteration in judgments. There are two lines of evidence for this claim. First, there is evidence that ego depletion does not merely cause a failure to inhibit a response; it changes patterns of choice for future times. Ego depleted subjects not only choose more tempting (over more worthy) options when they are immediately available, they also choose that the same tempting goods be delivered days later (Wang et al. 2010). This pattern of choices is difficult to explain unless we suppose that subjects have experienced some kind of alteration in their judgments (Levy 2011). Perhaps we can understand agents being compelled by their desires, and against their better judgments, to choose immediately available goods, but it is hard to see how they could be compelled against their own judgments to choose goods to be delivered at some later time. Such future-oriented choices seem to be expressions of subjects’ judgments, if anything is (all this is, of course, compatible with judgment-shift being *caused* by desires; the claim is that the best explanation of behavior does not involve action contrary to the agent’s concurrent better judgment).

The second piece of evidence that loss of control in the ego depletion paradigm is mediated by judgment change comes from studies probing the effects of resource depletion on attitudes. Wheeler et al. (2007) found that depleted subjects were significantly more persuaded by weak arguments than were controls. Similarly, Burkley (2008) found that resisting persuasion was ego depleting, and that ego depleted subjects were more easily persuadable.

I suggest that we can best interpret these findings within the error prediction framework. A counterattitudinal argument, even a weak argument, presents inputs that are discordant with the predictions generated by the model modulating bottom-up input. Ego depletion may turn up the gain on these inputs, or weaken top-down modulation. The result is pressure to revise the model. The depleted person, like the addict, has a belief landscape that is structured by rival attractors. For the tempted person (whether the temptation is drugs, chocolate, or simply the temptation of ceasing to engage in effortful activity), the attractions of the temptation are well-known; the model that entails or constitutes the judgment that giving in is best, all things considered, is easily available for recall, and the temptation makes it highly salient. Further, we can expect the temptation to be ‘wanted’, and therefore to motivate the organism to take steps to minimize the prediction error. The prediction-error generated by an argument, or by the counterattitudinal affordances of the tempting good, can be attenuated by adopting the model; the system is therefore perturbed and may settle into an equilibrium around the rival attractor.

As in the case of the addict, however, there are rival pressures that may ensure that the new equilibrium is not stable over the medium term. Once the person is no longer under the immediate influence of the cue that generates the error, a rival attractor becomes more salient. Moreover, this attractor does a better job of minimizing the current prediction error than the model that confers value on the temptation, by explaining the attractions of health, of savings, or what have you (further, it also does a good job of explaining away the attractions of the rival model). The result is a cue-dependent oscillation in states, just as we see in addiction.

The picture just sketched constitutes relatively weak and relatively indirect evidence for the account of cue-driven belief oscillation characteristic of addiction. It is worth emphasizing, however, that both the loss of control seen in addiction and ordinary weakness of will involve reason-responsive behavior; behavior, that is, that seems to be under the control of doxastic states (as Austin (1979: 198) notes, we may “succumb to temptation with calm and even with finesse”). Given that this is the case, it seems that only a view that explains how we can be subject, in both cases, to oscillations in doxastic states has a hope of explaining the loss of control we see in both kinds of cases. The view sketched here has that great advantage.

## Conclusion

I have argued that the best explanation for some of the behaviour of addicts postulates oscillation between all things considered judgments with conflicting contents. I have suggested that (admittedly controversial, and still somewhat speculative) evidence from cognitive neuroscience and from social psychology supports the view I have put forward. On this view, addicts experience a cue-driven oscillation between judgments, transitioning from judging that they ought to abstain from drugs to judging they ought to consume (perhaps just on this occasion) under the influence of cues predictive of drug availability, because these cues trigger large prediction errors. Since addicts can attenuate the prediction error most easily, at least in very many cases, by altering their judgment of the value of consumption, they rapidly come to see the drugs as choiceworthy. But the state is not stable: once cues predictive of reward are removed, or their salience is attenuated by satiation, the costs (including the opportunity costs) of addiction become more salient. Once again, there is a mismatch between top-down model and inputs, and the addict revises her judgment to attenuate the error.

I want to conclude with a few brief reflections on whether the account is best understood, as I suggested in the introduction, as a story involving a loss of control. It doesn’t involve loss of control as that phrase is normally cashed out: the behaviour remains reasons-responsive and instrumentally rational (it was precisely these facts that motivated me to look for an explanation in terms of judgment shift in the first place). Nevertheless, there seem to be good grounds for thinking of the judgment oscillation as involving a loss of control over beliefs.

Notice, first, that the change of mind is not in response to evidence; or, at least, it is not in response to evidence that the person had not already considered in forming the contrary judgment. She was already well aware of the attractions of drugs. It was not learning anything new that prompted the change in judgment; rather, it was the workings of subpersonal mechanisms, in ways that she would not endorse were she not under the distorting influence of the cue. Second, in addiction the relevant valuational mechanisms are distorted by the influence of substances that in one way or another directly influence the currency that is supposed to track value, bypassing normal valuational pathways. It is plausible to think that judgments caused by a dysfunctional valuation system are not judgments over which the person has any control, even in the suitably deflationary and compatibilist sense of control in which it is often said that reasons-responsive mechanisms are controlled by agents.

Matters are harder in the case of ordinary judgment shift caused by ego-depletion. These may be cases of systems functioning as they were designed; prompting a shift in behaviour in response to the rapidly diminishing returns of further effort. Whether it is appropriate to see these shifts as involving a loss of control, mediated by judgment shift, is a difficult question (once again, it is worth pointing out that the alteration in judgment may not be in response to evidence that the person had not already taken into account in forming the initial, conflicting, judgment). One reason to think that the person experiences a loss of control over judgment formation is that in these circumstances reasons-responsiveness seems to be compromised, inasmuch as subjects find weak arguments significantly more convincing than matched controls.

These facts provide us with some grounds for thinking that ordinary weakness of will may often involve a loss of control mediated by judgment shift, and that addicts who find themselves consuming against their prior considered judgment that they ought to refrain typically have lost control in this manner. Whether this is an account of loss of control that can do the kind of work such accounts are commonly called upon to play (e.g., excusing the agent for wrongdoing) I leave to another day.

## Footnotes

1 It should be noted that there is an ongoing debate whether delusions are really beliefs, or some other kind of attitude instead. Opponents of the view that delusions are (full blown) beliefs point to their relative lack of integration with other states and the fact that sufferers often lack some of the dispositions we associate with belief possession. They also cite reports by sufferers indicating that at least some of them seem to concede that their delusions are not quite full-blown beliefs (see Bortolotti 2009 for discussion). Few theorists deny, however, that delusions are, or at very least cause, partially doxastic states. Though sufferers may fail to exhibit enough of the phenomenal and behavioral dispositions we associate with a full blown belief to count as possessing such a belief, they do possess enough of the relevant dispositions—including dispositions to verbal behavior, normally central to the dispositional stereotype—for it to be clear that delusions are, or typically cause, belief-like states. That is sufficient for my purposes here.

2 That is to say, the experience is made sense of—has a content assigned to it—very early in the processing hierarchy, by processes that are automatic and unconscious. Further, sense-making does not involve the mindreading faculty; hence even on a view like Carruthers’ (2011) it will not count as interpretive.

3 It may be that oscillation between conflicting doxastic states also occurs in classical delusions; this might help to explain the notorious mismatch between the content of the delusions to which patients are apparently committed and their behavior.

4 Corlett et al. 2007 note that rPFC activity is probably downstream from the prediction-error itself, reflecting inferences made in response to the prediction error.

5 It is important not to identify the motivations produced by prediction errors with the desires of folk psychology. There are two reasons. First, these motivational states are subpersonal and may lack any phenomenology. Second, these motivational states motivate doxastic update as much as action, but there is a plausible case for holding that the functional role of the desires of folk psychology is motivating action (in conjunction with relevant beliefs). Prediction errors may generate these kinds of desires when an action on the world better minimizes prediction error than a model update.
